# Nivolumab for advanced melanoma: pretreatment prognostic factors and early outcome markers during therapy

**DOI:** 10.18632/oncotarget.12677

**Published:** 2016-10-15

**Authors:** Yoshio Nakamura, Shigehisa Kitano, Akira Takahashi, Arata Tsutsumida, Kenjiro Namikawa, Keiji Tanese, Takayuki Abe, Takeru Funakoshi, Noboru Yamamoto, Masayuki Amagai, Naoya Yamazaki

**Affiliations:** ^1^ Department of Dermatologic Oncology, National Cancer Center Hospital, Tokyo, Japan; ^2^ Department of Experimental Therapeutics, National Cancer Center Hospital, Tokyo, Japan; ^3^ Department of Dermatology, Keio University School of Medicine, Tokyo, Japan; ^4^ Department of Preventive Medicine and Public Health, Biostatistics Unit at Clinical Translational Research Center, Keio University School of Medicine, Tokyo, Japan

**Keywords:** nivolumab, metastatic melanoma, absolute lymphocyte count, absolute neutrophil count, early markers for outcome

## Abstract

**Background:**

An anti-programmed cell death protein 1 monoclonal antibody, nivolumab, is one of the most effective drugs for advanced melanoma. Tumor cell-derived or immune cell-derived markers and clinical predictors such as serum lactate dehydrogenase (LDH) and cutaneous adverse events, have already been described as prognostic factors for advanced melanoma treated with nivolumab. We sought to identify further clinical predictors that can be determined in routine clinical practice.

**Methods:**

We retrospectively analyzed clinical findings of 98 consecutive patients with unresectable stage III or IV melanoma treated with nivolumab, at the National Cancer Center Hospital or at Keio University Hospital, in Tokyo, Japan, between July 2014 and July 2016. These patients had been administered nivolumab at a dose of 2mg/kg every 3 weeks.

**Results:**

As for pretreatment prognostic factors, ECOG performance status (PS) ≥1, maximum tumor diameters of ≥30mm, elevated LDH and elevated C-reactive protein were significantly associated with poor overall survival (OS) (hazard ratio [HR] 0.29 [*P*<0.001], HR 0.40 [*p*=0.003], HR 0.29 [*P*<0.001], HR 0.42 [*P*=0.004], respectively) on univariate analysis. Among these factors, PS and LDH were identified as independent variables by multivariate analysis. As for early markers examined during therapy, patients with absolute lymphocyte count (ALC) ≥ 1000/μl (Week3: HR 0.40 [*P*=0.004], Week6: HR 0.33 [*P*=0.001]) and absolute neutrophil count (ANC) <4000/μl (Week3: HR 0.46 [*P*=0.014], Week6: HR 0.51 [*P*=0.046]) had significantly better OS.

**Conclusion:**

ALC≥1000/μl and ANC<4000/μl during treatment appear to be early markers associated with OS. Nivolumab might have minimal efficacy in patients with a massive tumor burden.

## INTRODUCTION

Advanced melanoma has historically been regarded as a disease with a poor prognosis, with a median overall survival (OS) of 8-10 months and a 5-year survival rate of 10% [1]. In recent years, the emergence of new drugs, BRAF inhibitors and immune-checkpoint blockades, have resulted in remarkable advances in the treatment of advanced melanoma and have improved patient outcomes. According to the NCCN guidelines version 2.2016, anti-programmed cell death protein 1 (PD-1) antibodies (nivolumab and pembrolizumab) or the nivolumab/ipilimumab combination can serve as first-line immunotherapy for metastatic/unresectable melanoma, with single use of ipilimumab being recommended as second-line therapy. In Japan, nivolumab is the first immune-checkpoint inhibitor to be approved and has played the leading role in treatment of advanced melanoma since 2014. Recently, nivolumab was also approved for use in non-small-cell lung cancer [2, 3] and renal cell carcinoma [4].

Although immune-checkpoint inhibitors are used for all metastatic and unresectable melanomas, these agents are only effective for a portion of these malignancies and, above all, they are very expensive. Identifying biomarkers predicting efficacy and thereby allowing appropriate patients to be selected for these treatments is a crucial topic of ongoing research. Over the past several years, some biomarkers and clinical predictors of nivolumab efficacy in melanoma patients have been reported. The previously reported biomarkers were classified into two groups: tumor-derived immune biomarkers and immune cell-derived biomarkers. The former include tumor cell PD-L1 expression [5–7], tumor cell MHC-II expression [8], high tumor mutational load [9,10], and neoantigen [11]. The latter include the presence of CD8^+^ tumor-infiltrating lymphocytes (TILs) in tumor microenvironments [6, 12–14], increased PD-L1 expression on immune cells [15], no increase in peripheral-blood regulatory T cells, no decrease in antigen (NY-ESO-1, MART-1 and gp100) specific T cells [6], specific inflammation and IFN-γ-related mRNA-based signatures [16]. However, these biomarkers are not entirely reliable and their investigation is labor-intensive and thus impractical in daily clinical practice. On the other hand, predictors which are also routinely obtained clinical findings, such as serum lactate dehydrogenase (LDH) and cutaneous adverse events (AEs), have only been assessed by a small number of investigators [17, 18].

To enhance the efficacy of nivolumab, we analyzed simple predictors, focusing particularly on those easily obtained by routine laboratory testing.

## RESULTS

### Patient demographics

From July 2014 through July 2016, 98 patients were treated. Patient characteristics are summarized in Table 1.

**Table 1:** Demographic factors and baseline patient characteristics (n=54)

Factor	Category		*N* (%)
Age	<65		46(46.9)
	≥65		52(53.1)
	Median age (range)		66.5 (17-93)
Gender	Male		52(53.1)
	Female		46(46.9)
Stage	III		12 (12.2)
	IV		86 (87.7)
ECOG performance status	0		54 (55.1)
	1		38(38.8)
	2		5 (5.1)
	3		1 (1.0)
Primary site	Acral		17 (17.3)
	CSD		7 (7.1)
	Non-CSD		20 (20.4)
	Mucosal		36 (36.7)
	Others (Choroid)		11 (11.2)
	Unknown		7 (7.1)
Prior therapy	Chemotherapy	Systemic chemotherapy	24 (24.5)
		Transcatheter arterial chemoembolization	7 (7.1)
	Radiotherapy		18 (18.3)
	Immunotherapy	Adjuvant IFN-β (local injection)	12 (12.2)
		Dendritic cell therapy	4 (4.1)
		Ipilimumab	3 (3.1)
		PEG IFN-α 2b	1 (1.0)
	Others (Molecular therapy)		8 (8.2)
	None		28 (28.6)
Number of prior systemic therapies	0		28 (28.6)
	1		49 (50.0)
	2		13 (13.3)
	3		6 (6.1)
	4		2 (2.0)
	Median		1
Adverse events (Grade)	0		47(48.0)
	1		31(30.6)
	2		10(10.2)
	3		6 (6.1)
	4		4 (4.1)
	Median		1
Baseline MDT (mm)	<30		47 (49.5)
	≥30		48 (50.5)
	NA		3
	Median (Range)		30.3 (5-130)
Baseline LDH (IU/L) ULN=229	<230		48 (49.0)
	≥230		50 (51.0)
	Median (Range)		231.5 (137−2266)
Baseline CRP (mg/dl) ULN=0.29	<0.30		46 (48.4)
	≥0.30		49 (51.6)
	NA		3
	Median (Range)		0.31(0.01-12.7)
Baseline WBC count (×10^3^/μl) ULN=8.59	<8.60		90 (87.8)
	≥8.60		8 (12.2)
	Median (Range)		5.58 (2.4-15.9)
Baseline ANC (×10^3^/μl)	<4.0		53 (52)
	≥4.0		45 (48)
	Median (Range)		3.88(1.44-13.52)
Baseline ALC (×10^3^/μl)	<1.0		33 (33.7)
	≥1.0		65 (66.3)
	Median (Range)		1.17 (0.23-2.95)
Baseline AMC (×10^3^/μl)	<0.3		32 (32.7)
	≥0.3		66 (67.3)
	Median (Range)		0.35 (0.14-0.98)
Baseline AEC (×10^3^/μl)	<0.2		62 (74)
	≥0.2		36 (26)
	Median (Range)		0.12 (0.00-0.75)

ECOG, Eastern Cooperative Oncology Group; CSD, chronically sun-damaged (skin); LDH, lactate dehydrogenase; ULN, upper limit of normal; IFN, interferon; CRP, C-reactive protein; WBC, white blood cell; ANC, absolute neutrophil count; ALC, absolute lymphocyte count; AMC, absolute monocyte count; AEC, absolute eosinophil count; MDT, maximum diameter of tumors; NA, not available.

The median patient age was 66.5 years (range, 17-93) and 52 (53.1%) patients were men. PS was 0 to 1 in 93.9% (*N*=92) of patients, and 87.7% (N=86) of patients had stage IV disease. While acral type is relatively common in Asia, more patients with mucosal melanoma participated in this study (Acral: 17.3%, CSD (chronically sun-damaged): 7.1%, Non-CSD: 20.4%, Mucosal: 36.7%, Others: 11.2% [10 patients *had a choroidal origin, and 1 patient had a lung origin*], unknown: 7.1%). Overall, 49% (*N*=48) of patients had elevated LDH at baseline.

Prior treatments had been administered to 71.4% patients (*N*=70). Among them, 24.5% (*N*=24) had received prior cytotoxic systemic chemotherapy (mainly Dacarbazine alone and combinations containing Dacarbazine), and 7.1% (*N*=7) had undergone transcatheter arterial chemoembolization. Radiotherapy had been administered to 18.4% (*N*=18) and some patients had also received immunotherapy (adjuvant IFN-β local injection: 12.2%, dendritic cell therapy 4.1%, Ipilimumab: 3.1%, PEG IFN-α 2b:1%). Numbers of prior systemic therapies ranged one to four (Median: 1). Numbers of organs with metastatic disease ranged from zero to seven (Median: 2). Maximum diameters of tumors (MDT) ranged from 6 to 130 mm (Median: 30.3mm). The MDT sites varied (27 liver, 24 lymph node, 16 lung, 8 bone, 4 subcutaneous, 4 gastrointestinal tract, 2 adrenal grand, 2 pleura, 2 nasal cavity, 1 brain, 1 spleen, 1 gallbladder, 1 pancreas, 1 intestinal membrane and 1 muscle. 3 patients had unmeasurable tumors.)

Baseline serum LDH values ranged from 137-2266 (/μl) and the median was 231.5. LDH was elevated (upper limit of normal [ULN]: 229) in 51% (*N*=50) of patients. Baseline CRP ranged from 0.01-12.7 (mg/dl) and the median was 0.31. CRP (ULN: 0.29) was elevated in 51.6% (*N*=49, 3/98 patients were not tested) of patients.

### Immune-related adverse events

In total, 52% (N=51) of patients experienced immune-related adverse events (irAEs). Most had grade (G) 1 AE (*N*=31). Major AEs included vitiligo (*N*=13), hypothyroidism (*N*=11), pruritis (*N*=10), rash (*N*=7) and malaise (*N*=5). Most of the AEs were mild (G1). In total, 20 patients had AEs of G2 or greater severity (G2: 10, G3: 6, G4: 4). G3 AEs included 2 Stevens Johnson Syndromes, 1 adrenal insufficiency, 1 diarrhea, 1 uveitis and 1 decreased platelet count. G4 AEs included 2 elevated CKs and 2 hyperglycemias.

### Clinical responses and survival

The best response rate was 22.4% (2 CRs and 20 PRs) and the overall response was 19.3% (2 CRs and 17 PRs). Two patients experienced PD before PR. Three patients experienced SD prior to PR. Two patients progressed to PD after having once been evaluated as showing PR. Five patients also showed PD after having once been given an evaluation of SD. Most remaining patients showed no change in response after the first evaluation or died after the confirmation of PD.

Median OS was 13.0 months (95%CI 5.7 to 20.3). Outcomes are summarized in Table 2.

**Table 2:** Clinical Responses and Survival

Best response	CR	2/98	2.0% (95% CI: 0.3-7.2%)
	PR	20/98	20.4% (95% CI: 12.9-29.7%)
	SD	24/98	24.5% (95% CI: 16.4-34.2%)
	PD	52/98	53.1% (95% CI: 42.7-63.2%)
Overall response	CR	2/98	2.0% (95% CI: 0.3-7.2%)
	PR	17/98	17.3% (95% CI: 10.4-26.3%)
	SD	16/98	16.3% (95% CI: 9.6-25.2%)
	PD	63/98	64.3% (95% CI: 54.0-73.7%)
OS (Months)	Median		13.0 (95% CI: 5.7-20.3)

### Baseline biomarker evaluation

**Figure 1:**
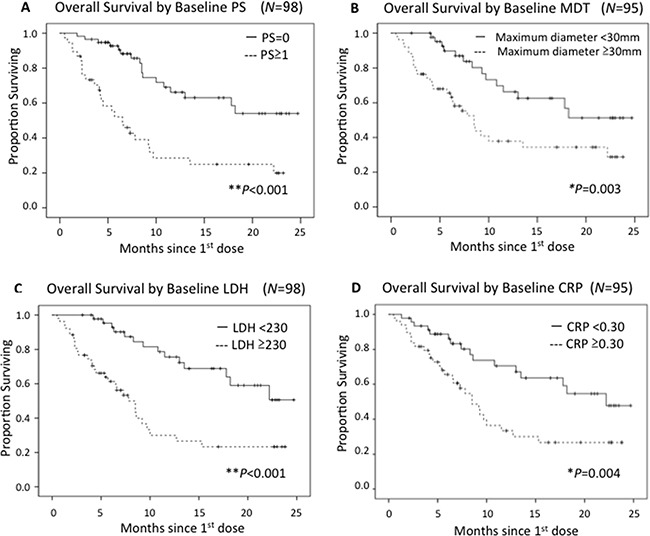
Kaplan-Meier curves in terms of OS by each prognostic factor PS≧1 **A.**, MDT **B.**, elevated LDH **C.** and elevated CRP **D.** were associated with significantly poorer outcomes.

Examination of the baseline findings revealed that patients with PS=0 had a significantly longer OS than those with PS≥1 (54 patients vs 44; HR 0.29, 95% CI 0.16-0.53, *P*<0.001) (Figure 1A). Since there were only 6 patients with PS≥2, the difference between PS≤1 and PS≥2 was not significant (*P*=0.190). Tumor size was also significantly associated with OS (MDT<30mm [*N*=47] vs ≥30mm [*N*=48]; HR 0.40, 95%CI 0.21-0.75, *P*=0.03) (Figure 1B). Sex, age, stage III or IV, primary site, prior therapy, and number of prior systemic therapies, likewise, showed no significant differences. Normal, versus elevated, baseline serum LDH and CRP also correlated significantly with longer OS (LDH: 48 patients vs 45; HR 0.29, 95%CI 0.15−0.55, *P*<0.001, CRP: 46 vs 49; HR 0.42, 95%CI 0.23-0.77, *P*=0.004) (Figure 1C, 1D).

**Figure 2:**
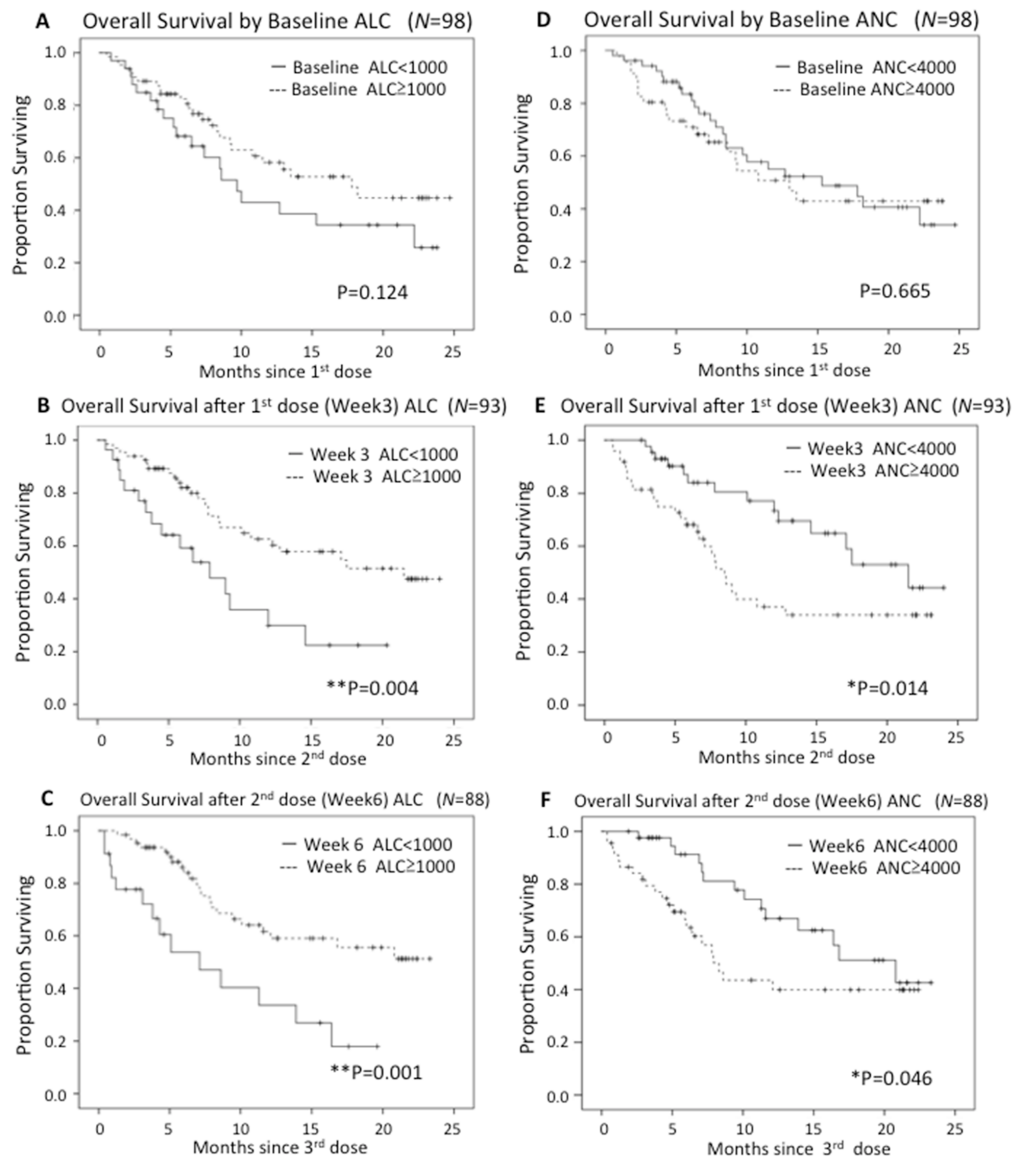
Kaplan-Meier curves in terms of OS by ALC and ANC ALC and ANC after the 1^st^ nivolumab dose correlated with survival.

Finally, we investigated whether baseline peripheral blood test values might be related to outcomes. We analyzed whether the white blood cell count, as well as ALC, ANC, AMC or AEC, showed any correlation with OS. Only non-significant trends suggesting relationships between baseline values and outcome were detected (Figure 2A, 2D and Supplementary Figure A, B).

### Early markers of outcomes during therapy

We explored clinical data to identify early predictors of outcome during treatment.

**Figure 3:**
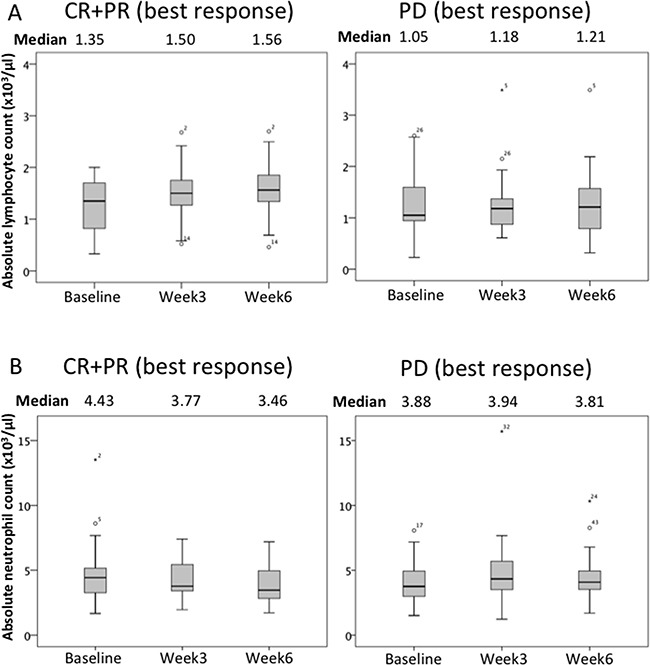
Changes in the ALC and the ANC of responsive (CR+PR) and non-responsive (PD) patients receiving nivolumab are shown Median ALC increased slightly as the therapy proceeded. The median ALC of responsive patients was higher than that of non-responsive patients. The median ANC of the responsive subgroup decreased during the course of therapy. The ANC of non-responsive patients was essentially unchanged.

ALC and ANC changes in responsive (CR+PR; best response) and non-responsive (PD; best response) patient subgroups during nivolumab therapy are shown in Figure 3. Median ALC increased slightly during the course of therapy in both groups. ALC values were clearly higher in responsive than in non-responsive patients. The median ANC of the responsive subgroup was decreased during the course of therapy. On the other hand, the ANC of the non-responsive patients was essentially unchanged. We stratified patients into two ALC groups based on a cut-off value of ≥1000/μL (high ALC) vs <1000/μL (low ALC). We also stratified Kaplan-Meier survival curves based on the ALCs at baseline and after the 1^st^ and 2^nd^ nivolumab doses (Week 3 and Week 6 after treatment initiation), as shown in Figure 2A-C. While baseline ALC was not significantly associated with OS (HR 0.63, 95%CI 0.35-1.14 *P*=0.124), ALC during nivolumab therapy showed a clear and significant association with OS (Week3: HR 0.40, 95%CI 0.21-0.77, *P*=0.004; Week6: HR 0.33, 95%CI 0.17-0.65, *P*=0.001).

We also stratified patients into high and low ANC, AMC and AEC groups during treatment. While there was no significant difference between high ANC (≥4000μL) and low ANC (<4000/μL) cases at baseline (HR 0.88, 95%CI 0.49-1.58, *P*=0.665), ANC during nivolumab therapy showed a significant association with OS (Week3: HR 0.46, 95%CI 0.24-0.87, *P*=0.014, Week6: HR 0.51, 95%CI 0.26-1.00, *P*=0.046). Kaplan-Meier survival curves based on ANCs are presented in Figure 2D-F.

Similarly, high AEC at Week 3 was significantly associated with better OS while there was no significant difference at Baseline or at Week 6 (≥100μL vs <100μL, Baseline; HR 0.77, 95%CI 0.41-1.45, *p*=0.418, Week3; HR 0.49, 95%CI 0.27-0.91, *p*=0.024, Week6; HR 0.55, 95%CI 0.28-1.05, *p*=0.068). (Supplementary Figure E-G)

According to these findings, high ALC and low ANC during treatment appear to be associated with a good prognosis. High AEC during treatment also suggestedbetter OS. Associations of WBC counts and AMC during treatment with outcome showed only non-significant trends. Univariate conditional survival analysis evaluating relationships between OS and laboratory data at each time point (baseline [Week 0], week 3, and week 6 after initial nivolumab dose) is summarized in Table 3.

**Table 3:** Univariate conditional survival analysis evaluating relationships between OS and laboratory data at each time point (baseline [Week 0], week 3, and week 6 after initial nivolumab dose)

	Baseline (N=98)		Week 3 (N=93)		Week 6 (N=87)	
Factor	HR (95%CI),	*P*	HR (95%CI),	*P*	HR (95%CI),	*P*
LDH<230	0.29 (0.15-0.55),	**<0.001**	0.28(0.15-0.53),	**<0.001**	0.27(0.14-0.53),	**<0.001**
CRP<0.30	0.42 (0.23-0.77),	**0.004**	0.24(0.12-0.48),	**<0.001**	0.26(0.13-0.54),	**<0.001**
WBC<8600	1.06 (0.42-2.69),	0.907	0.60(0.29-1.23),	0.156	1.02(0.40-2.64),	0.963
ALC≥1000	0.63 (0.88-2.86),	0.124	0.40(0.21-0.77),	**0.004**	0.33(0.17-0.65),	**0.001**
ANC<4000	0.88(0.49-1.58),	0.665	0.46(0.24-0.87),	**0.014**	0.51(0.26-1.00),	0.046
AMC<300	0.72(0.38-1.37),	0.307	0.83(0.38-1.79),	0.631	1.57(0.78-3.15),	0.200
AEC≥100	0.77(0.41-1.45),	0.418	0.49(0.27-0.91),	**0.024**	0.55(0.28-1.05),	0.068

In addition, patients with AEs (of all grades) had significantly better OS (HR 0.54, 95%CI 0.30-0.99, *P*=0.043). Notably, the 13 patients experiencing only vitiligo had good outcomes (HR 0.15, 95%CI 0.04-0.63, *P*=0.003). (Supplementary Figure C, D)

## DISCUSSION

### Comparison with ipilimumab

To date, a number of potential markers for ipilimumab efficacy have been described. As for baseline peripheral blood test values, low LDH, CRP, AMC, and myeloid-derived suppressor cells (MDSC), as well as high AEC, CD4^+^CD25^+^FOXP3^+^-regulatory T cells frequencies, and relative lymphocyte counts are reportedly associated with a good prognosis [19–22]. As to early blood-based markers predicting responses to therapy, increased ALC [19, 23, 24], increased rate ALC to WBC [25], low neutrophil to lymphocyte ratio (NLR) [26], increased AEC [26], increased inducible co-stimulator [26, 27] on circulating CD4^+^ T cells, decreased MDSC [28, 29], increased Th12 cell inducibility [30], and melanoma markers on circulating cells (Melan-A, gp100, MAGE-3 and MIA) [31] have been reported.

As previously described, several potential biomarkers for nivolumab have been suggested in recent years [5–18]. As to routinely-obtained clinical findings, only cutaneous AEs and LDH have been reported [17, 18].

In this study, as in prior investigations of ipilimumab, LDH and CRP were shown to be baseline prognostic factors. Multivariate analysis of relationships between pretreatment levels of PS, MDT, CRP and LDH with OS are shown in Table 4A. PS and LDH were identified as independent variables, while MDT and CRP were not. MDT and CRP might simply reflect tumor burden or disease progression, in parallel with rising LDH. In addition, these pretreatment factors could simply reflect patients’ conditions, rather than nivolumab efficacy itself. If true, these results raise the possibility that nivolumab may not have sufficient efficacy in patients with a massive tumor burden.

**Table 4A:** Multivariate analysis of relationships of pretreatment baseline PS, MDT, CRP and LDH with OS (*N=93*)

Factor	HR (95%CI)	*P*
PS=0 (PS<1)	0.41(0.21-0.77),	**0.006**
MDT<30	0.59(0.31-1.15),	0.120
LDH<230	0.39(0.19-0.82),	**0.012**
CRP<0.30	0.90(0.45-1.99),	0.904

As to early markers for outcome, patients with high ALC and low ANC at week 3 and week 6 had significantly longer OS. Multivariate analysis of relationships between ALC and ANC at 3 and 6 weeks are shown in Table 4B. ALC and ANC were found to be independent variables. Good outcomes of patients with ALC≥1000 during treatment were likewise obtained in an ipilimumab study [19, 23, 24]. Though median ALC was also elevated in PD patients (ALC was increased as compared with baseline; CR+PR: 3W 13/22, 6W 15/22, PD: 3W 21/52, 6W 22/52), ALCs of non-responsive patients tended to be lower than those of the responsive subgroup (Figure 3).

**Table 4B:** Multivariate analysis of relationships of Week 3 (*N*=93) and Week 6 (*N*=88)

ALC and ANC with OS		
**Factor**	**HR (95%CI)**	***P***
Week 3 ALC≥1000	0.43(0.24-0.77),	**0.002**
Week 3 ANC<4000	0.65(0.39-1.08),	**0.007**
Week 6 ALC≥1000	0.33(0.19-0.58),	**0.001**
Week6 ANC<4000	0.82(0.48-1.38),	**0.042**

As for NLR, while baseline NLR was not significantly associated with OS, the patients with NLR≥4 at Week 3 tended to have poorer OS than those with lower NLR (comparison between NLR<4 and NLR≥4, Baseline: HR 0.65, 95%CI 0.36-1.17, *P*=0.147, Week3: HR 0.36, 95%CI 0.19-0.67, *P*= 0.001, Week6: HR 0.28, 95%CI 0.28-1.08, *P*=0.056) (Supplementary Figure H-J).

Our results appear to be consistent with those of studies examining early markers in patients receiving ipilimumab treatment.

### AEs and prognosis

AEs including vitiligo and rash were reported to be good prognostic factors for melanoma patients treated with nivolumab [17]. Similar results were obtained in this study.

### Delayed response

According to previous reports, some melanoma patients treated with ipilimumab experienced initial enlargement of tumor lesions, confirmed by biopsy to be attributable to inflammatory cell infiltrates or necrosis, followed by a subsequent decrease in tumor burden [32]. Such immune-related delayed clinical responses have also been observed in studies of nivolumab. One study of metastatic melanoma patients treated with nivolumab found that 10% (11 of 107 patients) experienced delayed responses [33]. However, in this study, only 2% (2 of 98 patients) experienced PD prior to PR, raising the possibility that the delayed response might be very limited in previously treated patients or limited to tumors arising from acral and mucosal sites, except in cases with CSD skin.

In addition, second or later evaluations yielded results not differing from those of the first, in most cases. Given these results and the major financial burden, we should be wary of continuing this treatment in patients initially evaluated as showing PD.

Though external validation is essential before hypothetical models can be applied in clinical practice, our preliminary results merit a large cohort analysis evaluating these factors in greater detail.

In conclusion, high ALC and low ANC after the 1^st^ nivolumab dose may serve as early markers associated with better OS in patients with advanced melanoma, based on our retrospective observations. Furthermore, PS, MDT, and CRP at baseline, along with the already established LDH, are potential prognostic markers for advanced melanoma cases. In addition, nivolumab appears to have an insufficient effect in patients with massive tumor burdens and, on rare occasion, a delayed response may occur in previously treated patients. Therefore, it might be worth considering the discontinuation of nivolumab administration in patients initially evaluated as showing PD, especially when such patients still have other treatment options. Further prospective study is warranted to assess these possibilities.

## MATERIALS AND METHODS

### Patients

We retrospectively analyzed all patients at the National Cancer Center Hospital (NCCH), Tokyo, Japan and Keio University Hospital, Tokyo, Japan, with advanced melanoma, treated between 2014 and 2016 using nivolumab, for whom outcomes could be evaluated. Eligibility criteria included unresectable stage III and IV melanoma, all of which had been histologically confirmed at the NCCH or Keio University. The patients who had been administered oral steroids were excluded considering the influences of these drugs on laboratory findings.

The items determined prior to treatment included age, sex, ECOG performance status (PS), stage, primary site, prior therapy, number of prior systemic therapies, number of organs with metastasis, maximum tumor diameters (in the event of lymph nodes harboring the largest tumors, the minor axis was measured) and peripheral blood tests (including serum LDH, C-reactive protein [CRP], absolute lymphocyte count [ALC], absolute neutrophil count [ANC], absolute monocyte count [AMC], absolute eosinophil count [AEC] and the ratios of the parameters to each other). Prior to each nivolumab administration, patients underwent repeat peripheral blood testing and AEs were evaluated. We continued to measure these parameters, during the treatment period, to determine whether they predicted outcomes.

### Treatment and response

Nivolumab was administered at 2mg/kg intravenously over 60 minutes every 3 weeks. This is the established dosing method for nivolumab covered by the national health insurance system of Japan. Patients continued this therapy until they were evaluated as showing progressive disease (PD) twice in succession, died or experienced unacceptable AEs. Patients who stopped nivolumab administration continued to be observed until death or until they were lost to follow-up.

Patients usually underwent radiographic imaging every 12 weeks and were evaluated for response by computed tomography (CT) according to the immune-related response evaluation criteria in solid tumors (irRESIST) criteria (version 1.1) [34, 35]. The response categories were complete response (CR), partial response (PR), stable disease (SD) and PD.

### Endpoint

The efficacy evaluation was based on survival rather than on progression free survival or best objective tumor response. The latter parameters were not considered to be appropriate for evaluating the actual benefits of nivolumab treatment. When baseline parameters were evaluated as prognostic factors, survival time was calculated as the period from the first dose of nivolumab to the date of death or the last documented follow-up.

When laboratory findings during treatment were evaluated as early markers, OS was defined based on the period from the date on which the patient underwent testing (3 and 6 weeks after starting treatment) until the date of death or last follow-up.

### Statistical analysis

Demographic factors and baseline patient characteristics of the study participants were summarized. The OS rate was estimated for each group with the Kaplan-Meier method. The log-rank test was used to compare survival between groups, and the hazard ratio (HR) and its 95% confidence interval (95% CI) were estimated employing Cox's proportional hazards model in the univariate analysis. Cox's proportional hazards models were also used for multivariate analyses. The conditional Cox regression model was used to evaluate the associations between early laboratory biomarkers after an initial dose of nivolumab and OS. For example, when evaluating the effect of a biomarker at Week 3, i.e. with the log conditional hazard function for given a survival time being greater than that at Week 3, the conditional Cox regression model was used. The 95% CI for proportions were estimated with the Clopper Pearson method.

The significance level for all tests was two-sided α = 0.05. All analyses were performed using the Statistical Package for Social Science (SPSS, Chicago, Ill) vesion23 for MAC.

## SUPPLEMENTARY MATERIALS FIGURES


