# Supplementary material for: Nivolumab for advanced melanoma: pretreatment prognostic factors and early outcome markers during therapy
Source: Oncotarget. 2016 Oct 15;7(47):77404–15. doi: 10.18632/oncotarget.12677 (PMC5363594; doi:10.18632/oncotarget.12677)
Supplement: Supplementary file 1 [file oncotarget-07-77404-s001.pdf]

Nivolumab for advanced melanoma: pretreatment prognostic factors and early outcome markers during therapy

SUPPLEMENTARY FIGURE

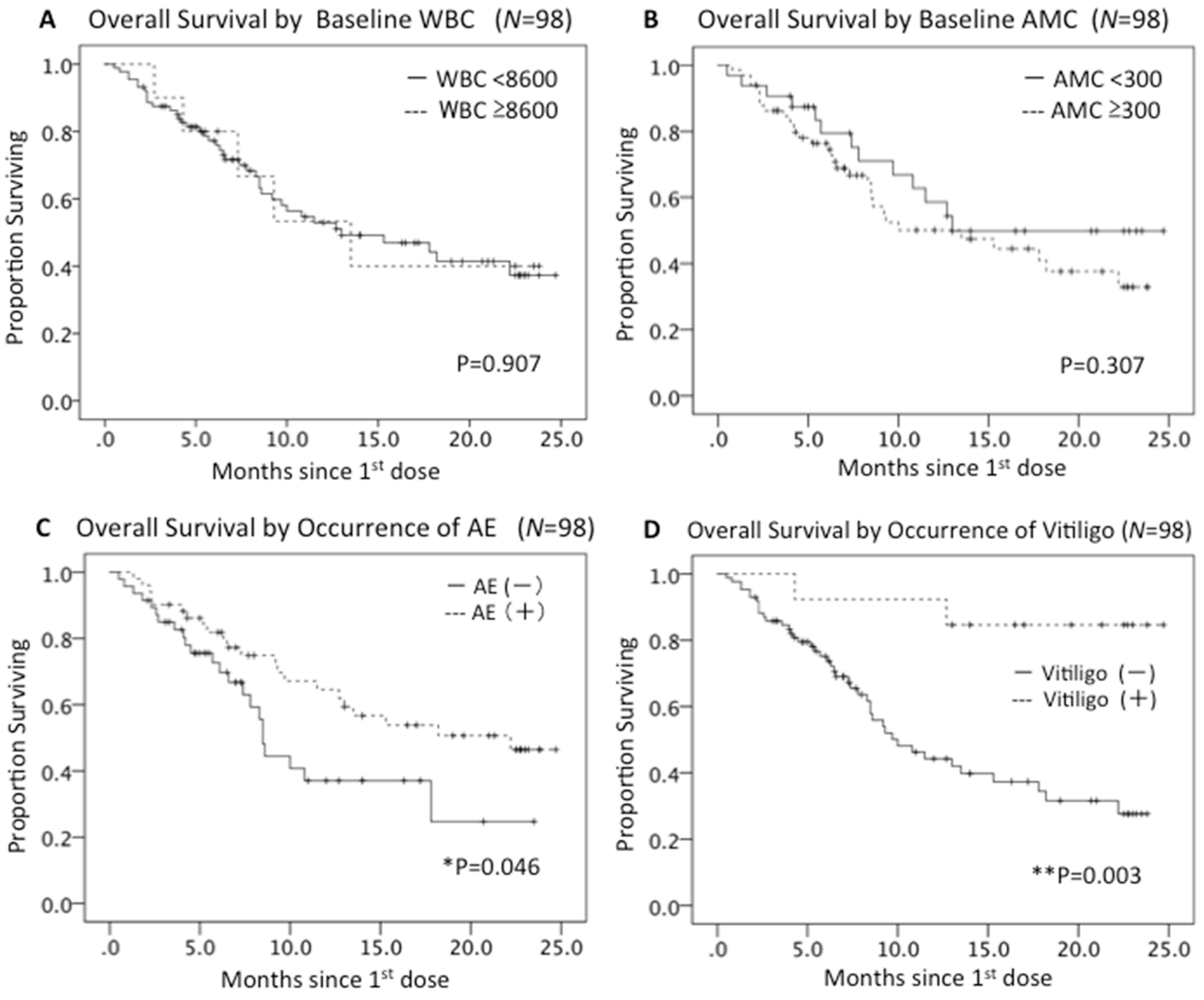

(Continued)

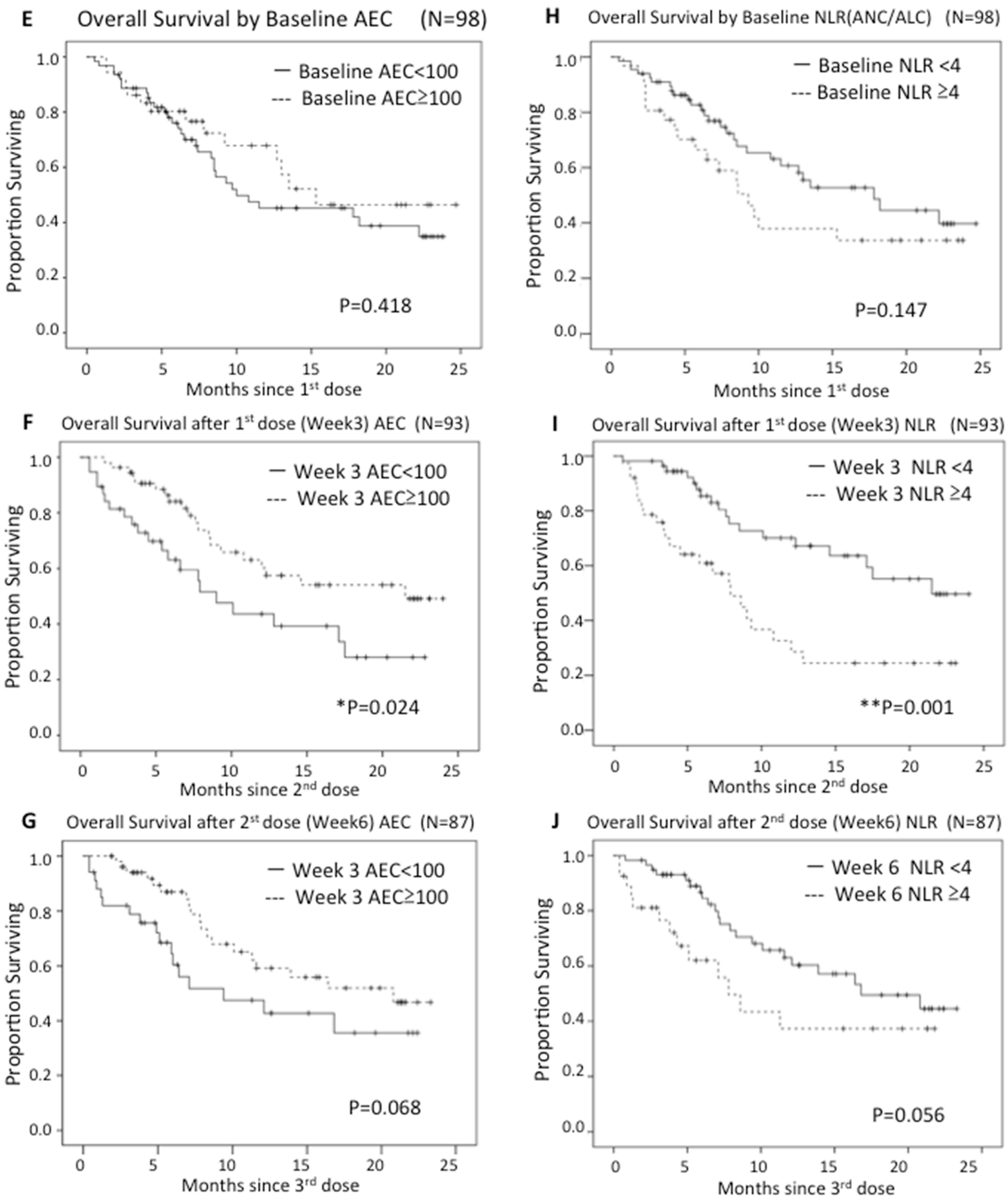

Supplementary Figure S1: Kaplan-Meier curves in terms of OS by each prognostic factor. Baseline WBC **A.**, AMC **B.**, AEC **E.**, and NLR **H.** were not associated with OS, significantly. Occurrence of AE **C.** especially vitiligo **D.** was significantly associated with better OS. AEC ≥100/μl **F.** and NLR <4 **I.** at Week 3 were associated with better OS significantly, while there was no significant difference at Week 6 **G, J.**
